# Spurious Presentation of Pulmonary Kaposi Sarcoma as Unresolved Pneumonia Led to Fatal Outcome

**DOI:** 10.1177/23247096231208996

**Published:** 2023-11-02

**Authors:** Baldeep Kaur Mann, Carlos D’Assumpcao, Lawrence Okumoto, Shatha Aboaid, Ayham Abooed, Everardo Cobos, Arash Heidari

**Affiliations:** 1 Kern Medical Center, Bakersfield, CA, USA

**Keywords:** Kaposi sarcoma, HHV-8, AIDS associated KS, Disseminated KS

## Abstract

Acquired immunodeficiency syndrome (AIDS)-associated Kaposi sarcoma (KS) is an angioproliferative neoplasia caused by infection with human herpesvirus 8 (HHV-8). It typically presents with mucocutaneous involvement, but it can be disseminated. Initial presentation with primarily pulmonary KS is rare. We present a case of a 32-year-old male with untreated human immunodeficiency virus (HIV) diagnosed 1 year before presentation who developed progressively worsening cough and shortness of breath for 6 months. He was hospitalized twice and treated for unresolved pneumonia in an outside hospital. The patient concomitantly developed purplish nodules on his face, then the upper trunk, back, chest, and thighs bilaterally that gradually increased in size and number. Histopathology findings from skin lesions were consistent for KS. Bronchoscopy found multiple erythematous plaques throughout the tracheobronchial tree with telangiectasias and inflammation suggestive of pulmonary KS. His imaging findings and positive serum HHV-8 polymerase chain reaction (PCR) were consistent with disseminated KS. He started antiretroviral therapy (ART) to treat his HIV infection, followed by liposomal doxorubicin chemotherapy. But both ART and chemotherapy were interrupted due to adherence and insurance issues. The patient was readmitted with acute respiratory failure requiring mechanical ventilation with multiple vasopressors that led to the patient’s demise. The late recognition of KS diagnosis and delayed treatment can lead to worse outcomes.

## Introduction

Kaposi sarcoma (KS), multicentric Castleman disease, primary effusion lymphoma, and KS herpesvirus (KSHV) inflammatory cytokine syndrome are the spectrum of KSHV-associated diseases that are found in immunodeficient individuals.^1^ Kaposi sarcoma is a multiorgan involving disease affecting the endothelial cells.^2^ Classic and iatrogenic KS presents as gradually worsening purplish macules, plaques, ulcers, and nodules with mucosal involvement.^2^ Acquired immunodeficiency syndrome (AIDS)-associated KS has been found to have more frequent gastrointestinal and pulmonary mucosal involvement with regression of the size of lesions on starting antiretroviral therapy (ART).^2^ At the onset of the AIDS epidemic, this newly discovered angiosarcomatous neoplasm associated with human herpesvirus 8 (HHV-8) frequently had pulmonary manifestations but decreased to a great extent in the post-ART era.^3^

We hereby present a case of fatal disseminated KS where the diagnosis was delayed for several months and then revealed with the help of cutaneous manifestations and a subsequent bronchoscopy. Unfortunately, the aggressive course of the disease led to acute respiratory failure and the patient’s demise.

## Case Presentation

A 32-year-old male with human immunodeficiency virus (HIV) was diagnosed 1 year before the presentation but was non-adherent to ART and presented to the emergency department for evaluation of right thigh pain.

**Figure1.**
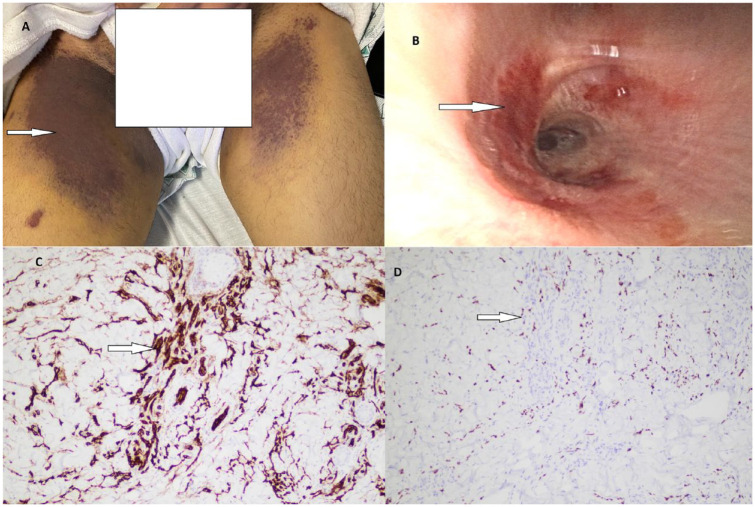
(A) Indurated non-pruritic purplish plaques on the medial aspect of thighs. (B) Erythematous mucous plaques discovered on bronchoscopy in the endobronchial area. (C) Histopathology of cutaneous skin lesion from the thigh showing endothelial cells stained immunohistochemically with diaminobenzidine anti-CD31 stain (10×). (D) Nuclear staining with HHV-8 stain (10×) from the thigh lesion biopsy. Abbreviations: HHV-8, human herpesvirus 8; CD31, cluster of differentiation 31.

The patient noticed the appearance of scattered purple-colored nodules on his face, then stomach, back, chest, and thighs for the last 2 months. The lesions on the thighs became progressively more painful, indurated, and larger in size (Figure 1A).

This was associated with a productive cough with yellowish sputum, dyspnea, pleuritic chest pain, and occasional non-bloody emesis due to repeated bouts of coughing for the last 6 months, which worsened over the last 3 to 4 weeks. One month before his presentation, he was hospitalized twice at another hospital for malaise, dyspnea, and cough with a diagnosis of community-acquired pneumonia, presumptive *Pneumocystis jirovecii*-induced (PCP) and treated with sulfamethoxazole/trimethoprim without improvement. The work-up during prior hospitalization, including viral nasal respiratory biofire panel, sputum *Mycobacterium tuberculosis* (MTB) PCR, sputum PCP-direct fluorescent antibody (DFA), urine legionella antigen, urine streptococcus pneumoniae antigen, urine histoplasma antigen, serum cryptococcal antigen, serum coccidioidomycosis antibody titers, and 1-3-beta-D-glucan, aspergillus galactomannan was unremarkable.

He had a history of smoking cigarettes, 1.8 pack years, which he quit 6 months ago, drinking 6 beers per week “on special occasions,” and smoked methamphetamine for 5 years which he quit 1 year ago. He was also experiencing homelessness and had multiple incarcerations in the past. He had a history of having sex with men, and his last partner was HIV-positive. He denied using pre-exposure HIV prophylaxis. Initial vital signs were blood pressure of 128/72 mm Hg, heart rate of 118 beats per minute, respiratory rate of 18 per minute, and temperature of 36.6°C, O_2_ saturation 98% at room air. His laboratory findings are shown in Table 1.

**Table 1.** Initial Laboratory Findings.

Name of the test	Value (normal range)
Hemoglobin	9.4 g/dL (13-17 g/dL)
White blood cells	6.1 × 103 mcL (4.5-11 × 10^3^ mcL)
Platelets	193 × 10^3^/μL (150-450 × 10^3^/μL)
Creatinine	1.02 mg/dL (0.67-1.17)
Alanine transaminase	67 unit/L (13-61 unit/L)
Aspartate aminotransferase	59 unit/L (15-37 unit/L)
Alkaline phosphatase	246 unit/L (45-117 unit/L)
C-reactive protein	2.79 mg/dL (<0.3 mg/dL)
Erythrocyte sedimentation ratio	35 mm/h (0-35 mm/h)
D-dimer	>5000 ng/mL (<500 ng/mL)
Procalcitonin	0.31 ng/mL (<0.5 ng/mL)
Absolute CD4+ cells	81 cells/mcL (490-1740 cells/mcL)
Serum lactate dehydrogenase (LDH)	177 unit/L (87-241 unit/L)
Serum total protein	6.6 g/dL (2.5-4.9 g/dL)
Serum HHV-8 DNA quantitative real-time PCR	1 659 788 copies/mL
Serum cryptococcal Ag	Not detected
Serum coccidioidomycosis immunodiffusion	IgM non-reactive. IgG non-reactive.
Serum coccidioidomycosis complement fixation titer	<1:2
Serum cytomegalovirus DNA quantitative PCR	385 International units/mL
Respiratory multiplex PCR (Biofire, BioMerieux, USA)	Positive for rhinovirus/enterovirus
Pleural LDH	184 unit/L
Pleural pH	7.54
Pleural protein	4300 mg/dL
Urine histoplasma Ag	Not detected
Toxoplasma Ab IgG	Non-reactive
(1-3)-beta-D-glucan assay	47 pg/mL

Abbreviations: LDH, lactate dehydrogenase; HHV-8, human herpesvirus 8; PCR, polymerase chain reaction.

**Figure 2.**
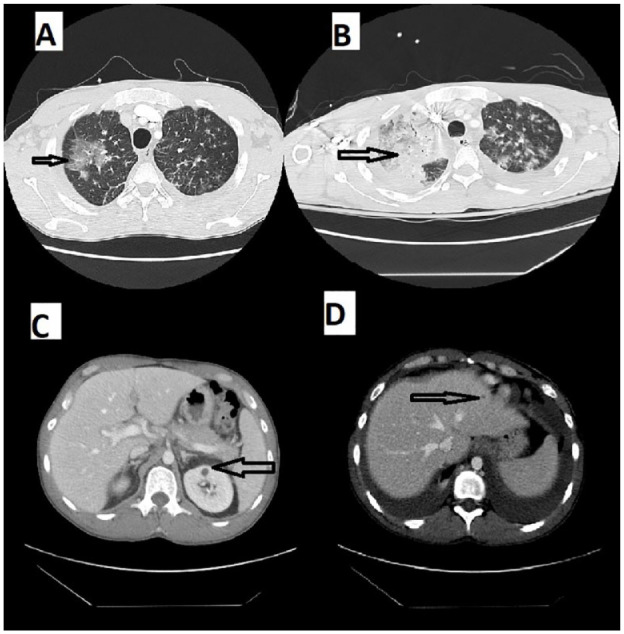
(A) CT chest indicating bilateral pleural effusion and scattered ground glass opacities. (B) Worsening ground glass opacities and alveolar infiltration on second hospitalization. (C) Left upper renal pole 1 cm hypodensity. (D) Subtle hepatic hypodensities worse in left hepatic lobe suggestive of disseminated KS. Abbreviations: CT, computed tomography; KS, Kaposi sarcoma.

The chest radiograph found bilateral lower lobe infiltrates and perihilar infiltrates. Patient was initially treated with vancomycin and piperacillin/tazobactam with concern for hospital-acquired pneumonia given recent hospitalization and sulfamethoxazole/trimethoprim (treatment dose) to cover for *PCP, fluconazole to cover endemic* coccidioidomycosis *and* cobicistat-boosted darunavir, emtricitabine, and tenofovir alafenamide. Computed tomography (CT) of the chest showed moderate basilar posterior partially loculated pleural effusions, worse on the left, extensive scattered ground glass infiltrates, worse on the right, and scattered alveolar consolidations without any pulmonary embolus with small bilateral hilar lymph nodes and slight pericardial effusion (Figure 2A). CT of the abdomen and pelvis showed a left upper renal pole 1 cm hypodensity and subtle hepatic hypodensities worse in the left hepatic lobe suggestive of disseminated KS (Figure 2C and D). During the course of hospitalization, the patient developed acute hypoxemic respiratory failure with increasing oxygen requirement, requiring high-flow nasal cannula with flow rate 30 to 60 liters/minute.

A diagnostic and therapeutic thoracentesis found dark bloody exudative pleural fluid with subsequent culture negative. Bronchoscopy revealed multiple erythematous plaques and lesions throughout the tracheobronchial tree with telangiectasias. These lesions were found in the trachea and down the bronchial tree. They were visually consistent with endobronchial KS (Figure 1B). The mucosa was inflamed and erythematous in the bilateral basal pulmonary segments. Bronchoalveolar lavage (BAL) and bronchial wash specimens ruled out infection (Table 2) and were negative for malignant cells. Endobronchial biopsy was avoided due to the risk of bleeding. Skin biopsy from his thigh and chest lesions stained positive by immunohistochemistry for co-expression of the endothelial marker cluster of differentiation 31 (CD31) and HHV-8 (Figure 1C and D), confirming cutaneous KS.

**Table 2.** BAL Work-Up Sent After Bronchoscopy.

BAL adenosine deaminase	19.4 unit/L
BAL MTB PCR	Not detected
BAL AFB smear	Negative
BAL AFB culture at 8 weeks	Negative
BAL RSV RNA, influenza A and B RNA, parainfluenza RNA	Not detected
BAL Aspergillus Ag	Not detected
BAL PCP DFA stain	Negative

Abbreviations: BAL, bronchoalveolar lavage; MTB, *Mycobacterium tuberculosis*; PCR, polymerase chain reaction; AFB, acid-fast bacilli; RSV, respiratory syncytial virus; PCP, *Pneumocystis jirovecii*; DFA, direct fluorescent antibody.

The positive serum HHV-8 PCR, supportive skin and bronchoscopic findings, and hypodense lesions in his kidney and liver were consistent with disseminated KS. Furthermore, the infectious work-up did not find another opportunistic infection. The patient was started on ART with cobicistat-boosted darunavir, emtricitabine, and tenofovir alafenamide followed by chemotherapy for KS with intravenous liposomal doxorubicin every 21 days.

He missed his second cycle of chemotherapy and presented with worsening shortness of breath and dyspnea on exertion after 1 month of the first cycle of chemotherapy. He quickly developed respiratory distress and shock and subsequently requiring endotracheal intubation and vasopressors. Repeat CT chest revealed worsening consolidation concerning for pneumonia or the progression of pulmonary KS (Figure 2B). He was placed on broad-spectrum antibiotics, including antifungal coverage and his ART was modified to a crushable regimen of dolutegravir/emtricitabine/tenofovir disoproxil fumarate. The chemotherapy was not given due to acute kidney injury requiring continuous renal replacement therapy. Infectious work-up of blood, sputum, urine, and repeat BAL were all negative on this admission as well. Despite maximal intensive supportive care, he continued to deteriorate and ultimately died.

## Discussion

Kaposi sarcoma was first described by Moritz Kaposi, an Austro-Hungarian dermatologist in 1872.^4^ Kaposi originally described as the classic or sporadic form that affects mostly elderly Mediterranean or Eastern European ancestry men and presents as dark purple-colored macules, patches, plaques or nodules on head and neck regions, upper and lower extremities, trunk, or oral mucosa. An endemic African form that presents mainly as lymphadenopathy in children was also found.^5^ In 1981, at the time of AIDS epidemic in New York, Alvin Friedman-Kien first described the epidemic or AIDS-associated KS in young homosexual men,^6,7^ a highly aggressive form of KS. Iatrogenic KS has also been described, particularly in post-organ transplant patients induced by immunosuppression.^8^ KS malignancy mainly affects endothelial cells and typically has cutaneous manifestations but may have visceral or mucosal involvement.^9^ AIDS KS is different from classic form, in that, it is more disseminated, rapidly progressive, and frequently fatal.^10^ AIDS -associated KS specifically affects viscera, including gastrointestinal tract and pulmonary.^11^

Our patient presented with several months of cough and dyspnea and was treated empirically by local physicians as presumptive *PCP* pneumonia due to his HIV diagnosis without any improvement. His progressively increasing number of skin lesions were suggestive of progression of KS. Bronchoscopy revealed erythematous plaques that were typically found in previously described endobronchial KS.^3^ Our patient had pink bronchoalveolar fluid with reddish telangiectatic mucosal plaques in the endobronchus (Figure 1B). His cutaneous lesions on the thighs were atypical presentation for KS and included symmetrical bilateral indurated slightly tender large plaques (Figure 1A). Induration was concerning for lymphatic KS. Immunostaining with CD31 and HHV-8 confirmed the diagnosis of cutaneous KS (Figure 1C and D).^12^ This case is a learning point for keeping the long-forgotten entity by the clinicians in post-ART era, endobronchial KS, as one of differential for unresolving pneumonia or respiratory failure in a HIV patient. The clinical manifestations may not be any different than infectious or neoplastic etiology. Ruling out other infectious etiologies before making diagnosis of KS is vital.^13^

Pulmonary KS has poor prognosis as it was depicted in this case and is essential to have it in the differential diagnosis for considering initiation of early treatment if suspected.^14^ The characteristic airway lesions ranged from peribronchovascular opacities, hemorrhagic pleural effusions, chylous effusions, and diffuse alveolar hemorrhage.^3^ Bronchoscopy with biopsy or BAL with PCR testing for HHV-8 could be utilized in suspected cases.^14^ Biopsy from the endobronchial lesion is generally avoided due to higher chances of bleeding.^10^ Early recognition and prompt treatment of KS at the appropriate time is crucial otherwise the patient may develop a fulminant irreversible disease course.

First-line therapy is to treat HIV in cases of AIDS-associated KS with the avoidance of systemic steroids. However, the patient may have flare-up of existing KS due to immune reconstitution inflammatory syndrome (IRIS),^10^ where systemic steroids may have a role. In cases of advanced disease, systemic chemotherapy should be initiated concomitantly or sequentially as changes in host immune system can lead to the persistence of KS despite control of viremia.^15^ But, there are no clear-cut criteria for starting systemic chemotherapy and the plan has to be individualized.^10^ Paclitaxel and pegylated liposomal doxorubicin (PLD) have been shown to decrease the size and number of lesions^16^ and is recommended as first-line cytotoxic agents for AIDS-related KS as per National Comprehensive Cancer Network (NCCN) guidelines, with response rates of about 50% to 60%.^16^

## Conclusion

Forty-two years after its initial description during the AIDS epidemic, AIDS-associated KS is still a cause of morbidity and mortality among persons with HIV. The cutaneous KS could be the clue for diagnosing dissemination forms, such as pulmonary KS, which has a poor outcome. Prompt treatment with ART and chemotherapy could prevent fulminant life-threatening entities and even mortality.
